# Structural basis for the ion selectivity of potassium-chloride cotransporter KCC4 revealed by cryo-EM titration

**DOI:** 10.52601/bpr.2025.240057

**Published:** 2026-06-30

**Authors:** Yuan Xie, Binming Han, Xin Tao, Fangjun Song, Cheng Zhao, Eric Delpire, Jingyuan Li, Shan Wu, Jiangtao Guo

**Affiliations:** 1 Department of Neurosurgery, Xijing Hospital, Fourth Military Medical University, Xi'an 710032, China; 2 School of Physics, Zhejiang University, Hangzhou 310058, China; 3 State Key Laboratory of Biocatalysis and Enzyme Engineering, Hubei Collaborative Innovation Center for Green Transformation of Bio-Resources, Hubei Key Laboratory of Industrial Biotechnology, School of Life Sciences, Hubei University, Wuhan 430062, China; 4 Department of Biophysics and Department of Neurology of the Fourth Affiliated Hospital, Zhejiang University School of Medicine, Hangzhou 310058, China; 5 Department of Anesthesiology, Vanderbilt University School of Medicine, Nashville, TN 37232, USA; 6 Nanhu Brain-Computer Interface Institute, Hangzhou 311100, China; 7 NHC and CAMS Key Laboratory of Medical Neurobiology, MOE Frontier Science Center for Brain Science and Brain-machine Integration, School of Brain Science and Brain Medicine, Zhejiang University, Hangzhou 310058, China; 8 State Key Laboratory of Plant Environmental Resilience, College of Life Sciences, Zhejiang University, Hangzhou 310058, China; 9 Cancer Center, Zhejiang University, Hangzhou 310058, China

**Keywords:** KCC4, Cryo-EM titration, Ions selectivity, Molecular dynamic simulation, Ions cotransport

## Abstract

Potassium-chloride cotransporters KCCs mediate the coupled, electroneutral cotransport of K^+^ and Cl^−^ across the membrane and are involved in important physiological processes such as cell volume regulation and γ-aminobutyric acid (GABA) and glycine-mediated inhibitory neurotransmission. Although structures of KCCs have been reported, the identification of ions bound in KCCs awaits experimental studies. Here using the cryo-electron microscopy (cryo-EM) titration methods, we present six structures of human KCC4 in different ion conditions at 2.38–2.58 Å resolutions. These structures, along with molecular dynamic simulations, allow us to assign one K^+^ and two Cl^−^ ions in the substrate-binding pocket. The K^+^ at S1 and Cl^−^ at the S2 site are tightly coupled in the binding and dissociation, suggesting that the Cl^−^ at S2 but not at S3 is the cotransported one. The S1 site provides coordination that largely matches the K^+^ dehydration radius and therefore displays higher selectivity to K^+^ over Na^+^. This study establishes the structural basis for the K^+^ selectivity of KCCs by the cryo-EM titration.

## INTRODUCTION

Ions not only serve as co-factors of many proteins such as enzymes but also are substrates of ion channels and transporters. Accurately identifying ions in protein structures and elucidating their working mechanisms are key tasks for the structural biology of ion-binding proteins. In comparison with protein peptides, ions bound in proteins usually have lower molecular weights, lower occupancies, and fewer structural features, which make it more challenging to identify ions in density maps. Several parameters can be used to assign which ion the density arises from in the map: (1) the shape and intensity of the density, (2) the chemical environment, *i*.*e*. ion coordination property, and (3) the ion composition in the protein sample. In X-ray crystallography, the anomalous signal of ions with high molecular weights generated during the X-ray diffraction provides additional evidence to assign these ions in protein structures (Pike *et al*. 2016). For example, in the potassium channel MthK, the K^+^ ions bound in the selectivity filter exhibit anomalous signals, which can be distinguished from water molecules and Na^+^ ions (Ye *et al*. 2010). Moreover, to study the structural basis of ion selectivity in potassium channels, people have developed the X-ray crystallographic titration method, which explores structure determination of the same potassium channel in different ion conditions and cross-validation of ion density from multiple data sets (Morais-Cabral *et al*. 2001; Sauer *et al*. 2013; Ye *et al*. 2010; Zhou and MacKinnon 2003). However, all these analyses of ion binding sites require high-resolution structures (at least 2.5 Å), which are difficult to obtain by X-ray crystallography of most ion channels and transporters due to their weak diffraction (Shi 2014).

The resolution evolution of single-particle cryo-EM significantly expedites the structure determination of ion channels and transporters (Cao *et al*. 2013; Kuhlbrandt 2014; Liao *et al*. 2013). In terms of ion identification, however, cryo-EM also has two disadvantages: (1) the overall resolution of most cryo-EM structures is 3.0–4.0 Å, which is still low for accurate assignment of ions (Earl *et al*. 2017), (2) heavy atoms generate no anomalous signal in the cryo-EM density map. Therefore, it’s more difficult to identify ions in the cryo-EM density map than that in the X-ray crystallography map. Previously, we have determined the cryo-EM structure of human potassium-chloride cotransporter KCC4 (PDB: 7D99, KCC4_7D99_), which can be readily pushed to high resolutions and therefore provides a good model for the ion identification by cryo-EM titration (Xie *et al*. 2020).

Potassium-chloride cotransporters KCC1-4 mediate the coupled, electroneutral cotransport of K^+^ and Cl^−^ across the plasma membrane and are critical for maintaining K^+^ and Cl^−^ homeostasis (Gamba 2005; Hebert *et al*. 2004). KCCs play important roles in various physiological processes, such as cell volume regulation (Delpire and Gagnon 2018; Lauf *et al*. 2001), auditory system function (Becker *et al*. 2003), and γ-aminobutyric acid (GABA) and glycine-mediated inhibitory neurotransmission (Hubner *et al*. 2001; Rivera *et al*. 1999). Dysfunction of KCCs leads to different human diseases, including epilepsy and Andermann syndrome, a rare neurodegenerative genetic disease associated with peripheral nerve abnormalities and various degrees of agenesis of the corpus callosum (Howard *et al*. 2002; Kahle *et al*. 2014; Saito *et al*. 2017; Stodberg *et al*. 2015). Besides, KCCs belong to the larger cation-chloride cotransporter (CCC) family, which also contains two sodium-potassium-chloride cotransporters (NKCCs) and one sodium-chloride cotransporter (NCC) (Arroyo *et al*. 2013; Russell 2000). Both NCC and NKCC2 are clinical targets of antihypertensive drugs (Chun *et al*. 2008; Orlov *et al*. 2014). CCCs share conserved overall architecture and ion binding sites, and elucidating their structures and ion transport mechanisms will guide the development of anti-epileptic and antihypertensive drugs (Chew *et al*. 2019; Chi *et al*. 2021a, b; Fan *et al*. 2023; Liu *et al*. 2019; Moseng *et al*. 2022; Nan *et al*. 2022; Neumann *et al*. 2022; Reid *et al*. 2020; Xie *et al*. 2020; Yang *et al*. 2020; Zhang *et al*. 2021; Zhao *et al*. 2022a, 2022b, 2024).

In the past several years, structures of KCCs have been reported by several groups. These structures reveal the overall structure, the autoinhibition by N-terminal peptide, and potential ions and ligands binding sites of KCCs (Chi *et al*. 2021a, b; Liu *et al*. 2019; Reid *et al*. 2020; Xie *et al*. 2020; Zhang *et al*. 2021; Zhao *et al*. 2022b). Due to different protein sample conditions and resolution limits, the assignment of ions bound in KCCs remains controversial. For example, the 3.7 Å resolution structure of the mouse KCC4 monomer reveals one K^+^ site and one Cl^−^ site (Reid *et al*. 2020), while in the 2.9 Å resolution structure of human KCC4 dimer (KCC4_7D99_) two Cl^−^ sites have been observed (Xie *et al*. 2020). Besides, in each study, assignments of ions in KCCs are based on the structures in the same ion condition, lacking cross-validation by structures of the same KCCs in different ion conditions. Under this circumstance, the experimental identification of ions in KCCs, and more importantly, the structural basis for the ion selectivity of KCCs, remain to be characterized. In this report, we have determined high-resolution cryo-EM structures of human KCC4 in different ion conditions. These structures, along with molecular dynamic (MD) simulation, elucidate the binding sites for different ions and the structural basis for ion selectivity in KCCs.

## RESULTS AND DISCUSSION

### High-resolution structures of KCC4 in different ion conditions

**Figure 1:**
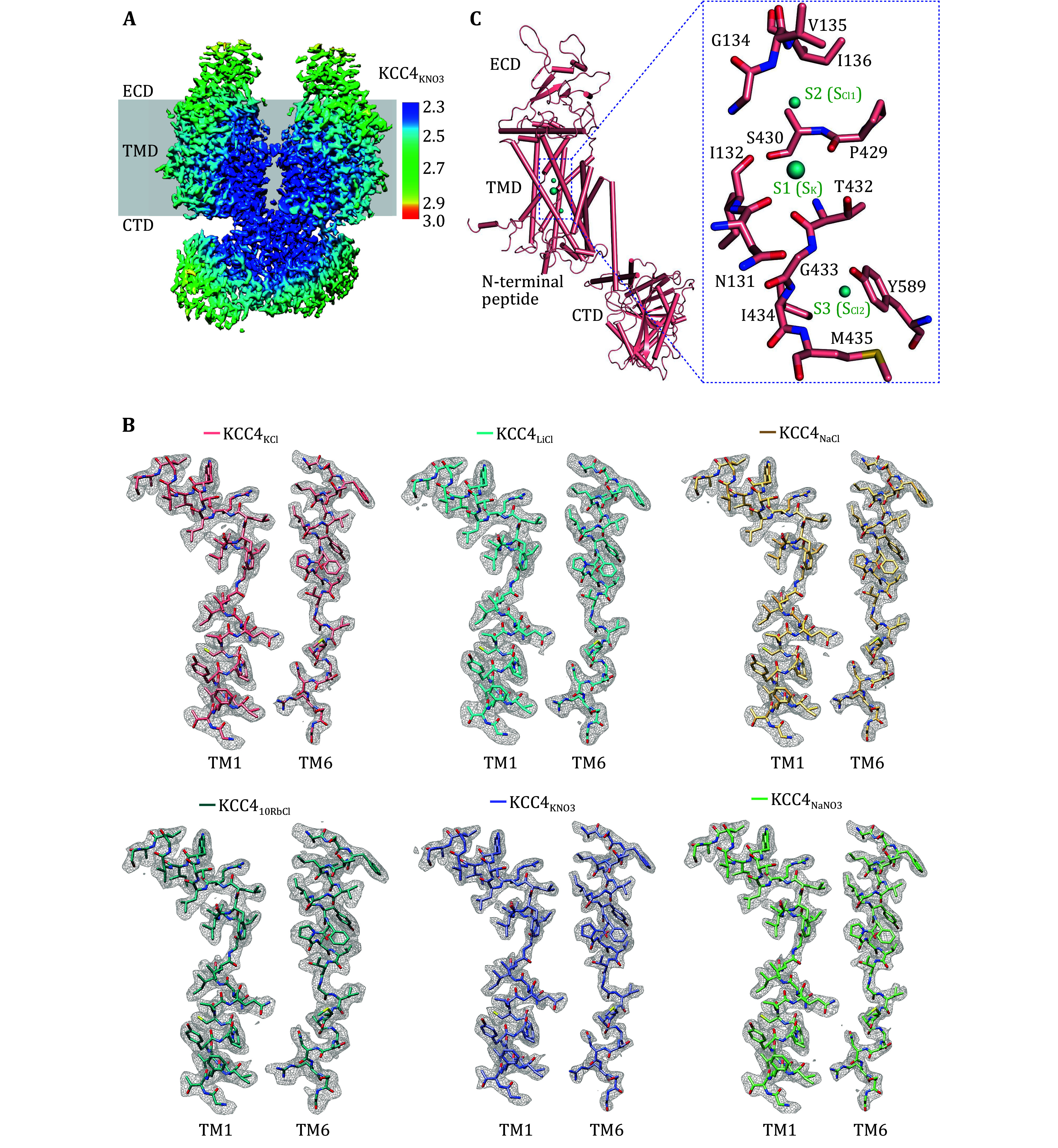
High-resolution structures of KCC4 in different ion conditions. **A** 3D reconstruction of KCC4_KNO3_ colored by local resolution. **B** Sample of EM maps at transmembrane helices 1 (TM1) and 6 (TM6) of KCC4 in different ion conditions at the contour level of 4 σ. **C** Cartoon representation of one KCC4_KCl_ subunit (left) and the zoom-in view of three potential ion binding sites S1–S3 (right). S1–S3 are equivalent to the three sites (S_K_, S_Cl1_, S_Cl2_) that we initially assigned in the KCC1_6KKR_ structure. The ions are shown as cyan spheres

To experimentally identify ions that bind in the substrate-binding pocket of KCC4, we have determined structures of KCC4 in 150 mmol/L LiCl (KCC4_LiCl_), in 150 mmol/L NaCl (KCC4_NaCl_), in 150 mmol/L KCl (KCC4_KCl_), in 140 mmol/L NaCl + 10 mmol/L RbCl (KCC4_10RbCl_), in 150 mmol/L KNO_3_ (KCC4_KNO3_), and in 150 mmol/L NaNO_3_ (KCC4_NaNO3_) at 2.38–2.58 Å resolutions (Fig. 1; supplementary Figs. S1–S6 and Table S1). These high-resolution structures allowed us to assign the ion as accurately as possible under current conditions. Like K^+^, the Li^+^, Na^+^, and Rb^+^ ions belong to the alkali metal group and therefore were included for comparative analysis. In addition, as an analogue of K^+^, Rb^+^ is often used as a substitute for K^+^ in functional assays and structural determination of K^+^ channels and transporters (Miyamoto *et al*. 1978; Morais-Cabral *et al*. 2001). By contrast, the anion NO_3_^−^ has a specific triangle-shaped density (Koropatkin *et al*. 2006), which would be distinguished from the sphere-shaped density of Cl^−^. In comparison with the 2.9 Å resolution KCC4_7D99_ we previously reported (Xie *et al*. 2020), these structures display the same domain-swapped dimeric assembly and inward-facing, autoinhibition conformation (Figs. 1A and 1C). No major conformational change was observed in different ion conditions, as revealed by the root mean square derivation (RMSD) of 0.26–0.38 Å over 891 Cα atoms within one subunit when KCC4_KCl_ is aligned with each of the rest five structures (supplementary Fig. S7). The improved resolutions allow us to not only accurately define the ions bound in the substrate-binding pocket but also resolve the conformation of both main chains and side chains of residues around these ions (Fig. 1C).

In our first 2.9 Å-resolution KCC1 structure (PDB: 6KKR, KCC1_6KKR_), we assigned one K^+^ and two Cl^−^ ions near the short Gly-containing loops in the middle of transmembrane helices TM1 and TM6 and named their sites as S_K_, S_Cl1,_ and S_Cl2_, respectively (Liu *et al*. 2019), which was cross-validated by other studies (Chew *et al*. 2019; Xie *et al*. 2020). In the 2.49 Å resolution KCC4_KCl_, the same three ion binding sites are also revealed (Fig. 1C). In this report, to perform rational assignments of ligands that bind at these three sites, we designate them as S1, S2, and S3, as shown in Fig. 1C. Meanwhile, to make strict comparisons of density levels of these three sites, we scaled all other five cryo-EM maps to the map of KCC4_KNO3_ which is of the highest resolution (Fig. 1A; supplementary Figs. S1–S6 and Table S1).

### Assignment of the Cl^−^ at S2 and S3 in KCC4_KCl_

**Figure 2:**
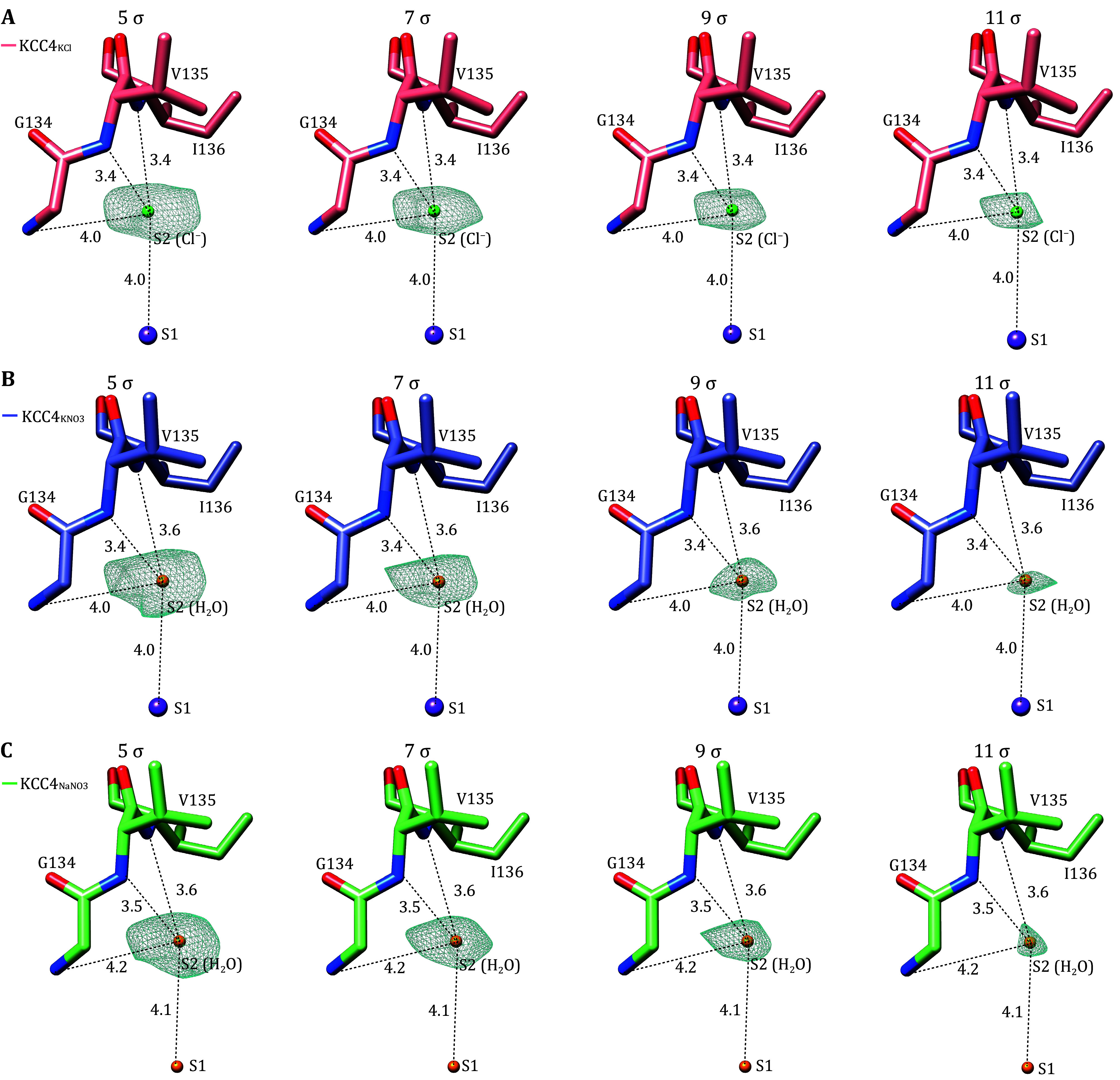
Assignment of Cl^−^ or H_2_O at S2 in KCC4_KCl_, KCC4_KNO3_, and KCC4_NaNO3_. **A** The Cl^−^ at the S2 site in the KCC4_KCl_ structure. **B** The H_2_O at the S2 site in the KCC4_KNO3_ structure. **C** The H_2_O at the S2 site in the KCC4_NaNO3_ structure. To perform the comparison, all maps are scaled and the densities are shown at the contour levels of 5 σ, 7 σ, 9 σ, and 11 σ. Dash lines show distances between Cl^−^ or H_2_O and their coordination residues in Å

**Figure 3:**
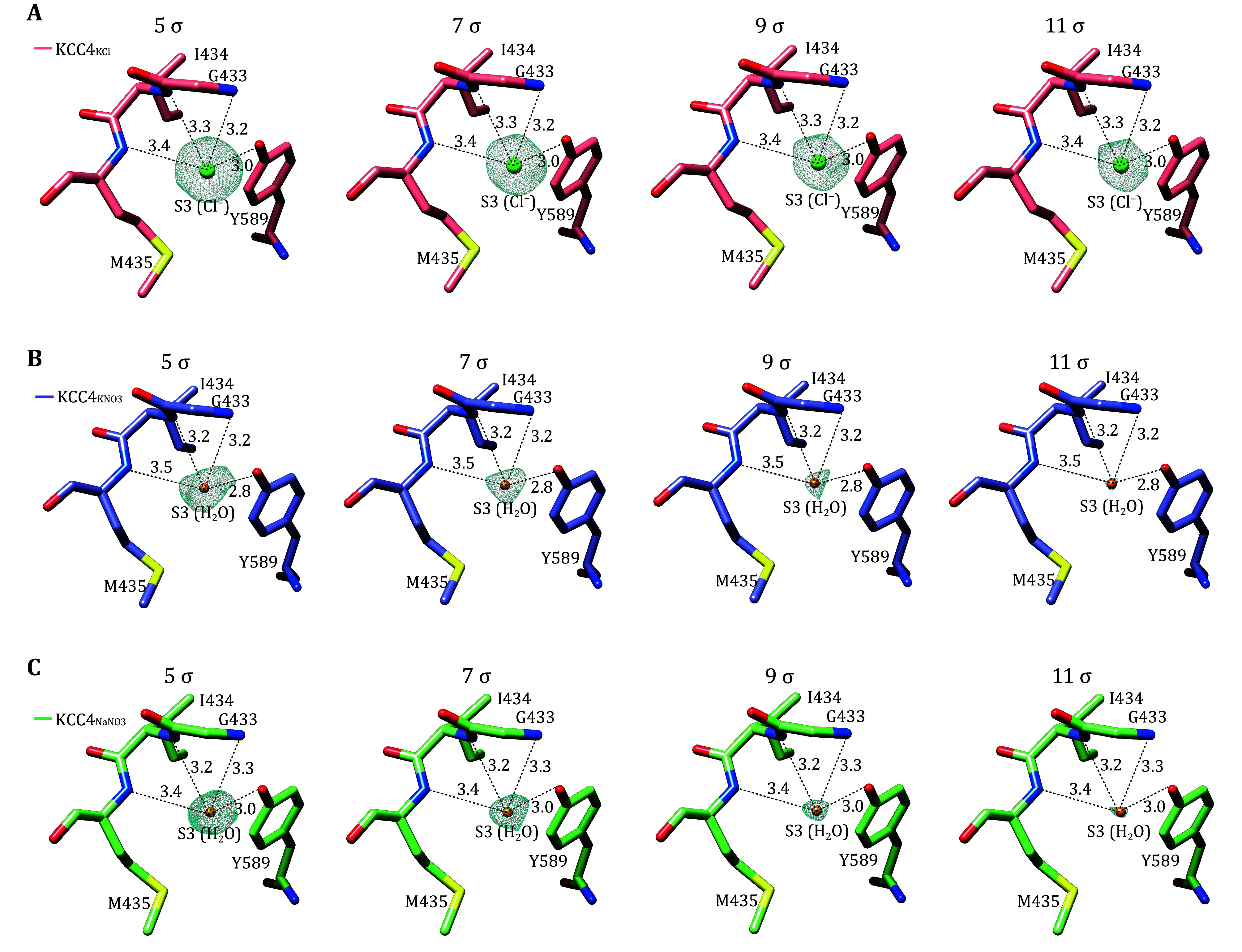
Assignment of Cl^−^ or H_2_O at S3 in KCC4_KCl_, KCC4_KNO3_, and KCC4_NaNO3_. **A** The Cl^−^ at the S3 site in the KCC4_KCl_ structure. **B** The H_2_O at the S3 site in the KCC4_KNO3_ structure. **C** The H_2_O at the S3 site in the KCC4_NaNO3_ structure. To perform the comparison, all maps are scaled and the densities are shown at the contour levels of 5 σ, 7 σ, 9 σ, and 11 σ. Dash lines show distances between Cl^−^ or H_2_O and their coordination residues in Å

We first examined the S2 and S3 sites in the structures of KCC4_KCl_, KCC4_KNO3,_ and KCC4_NaNO3_. S2 and S3 share similar chemical environments, where ions are mainly coordinated by three main chain amide groups, namely Gly134, Val135, and Ile136 for S2, and Gly433, Ile434, and Met435 for S3 (Figs. 2 and 3). Besides, the ion in S2 also interacts with the one in S1 (Fig. 2), whereas the Tyr589 side chain provides the fourth coordination for the ion in S3 (Fig. 3). These chemical environments of S2 and S3 favor anions or water molecules, but not cations.

We then checked the densities at the S2 and S3 sites in the maps of KCC4_KCl_, KCC4_KNO3,_ and KCC4_NaNO3_. In the KCC4_KCl_ map, densities at S2 and S3 sites are moderately strong, suggesting that Cl^−^ ions may bind there (Figs. 2A and 3A). By contrast, in the maps of KCC4_KNO3_ and KCC4_NaNO3_, densities at the S2 site are much weaker at the contour levels above 11 σ (Figs. 2B and 2C). Similar weaker densities are also observed at the S3 site in the maps of KCC4_KNO3_ and KCC4_NaNO3_ at the contour levels above 9 σ (Figs. 3B and 3C). The spherical shape and low intensity of densities at S2 and S3 indicate that water molecules but not ordered NO_3_^−^, the only anion in the protein sample, bind at the two sites in KCC4_KNO3_ and KCC4_NaNO3_ (Figs. 2B, 2C, 3B, and 3C), because NO_3_^−^ exhibits a triangle-shaped density, as in the 1.5 Å resolution structure of the nitrate-specific receptor NrtA (PDB: 2G29) (Koropatkin *et al*. 2006). Thus, the different intensities at S2 and S3 sites between the KCC4_KCl_ map and KCC4_KNO3_ or KCC4_NaNO3_ map confirm that Cl^−^ ions bind at S2 and S3 in KCC4_KCl_ (Figs. 2A and 3A), similar to the Cl^−^ ion bound in the prokaryotic Cl^−^/H^+^ transporter StClC (Dutzler *et al*. 2002). In the absence of Cl^−^ in KCC4_KNO3_ and KCC4_NaNO3_, water molecules likely occupy these two sites (Figs. 2B, 2C, 3B, and 3C).

### Assignment of the K^+^ at S1 in KCC4_KCl_ and KCC4_KNO3_

**Figure 4:**
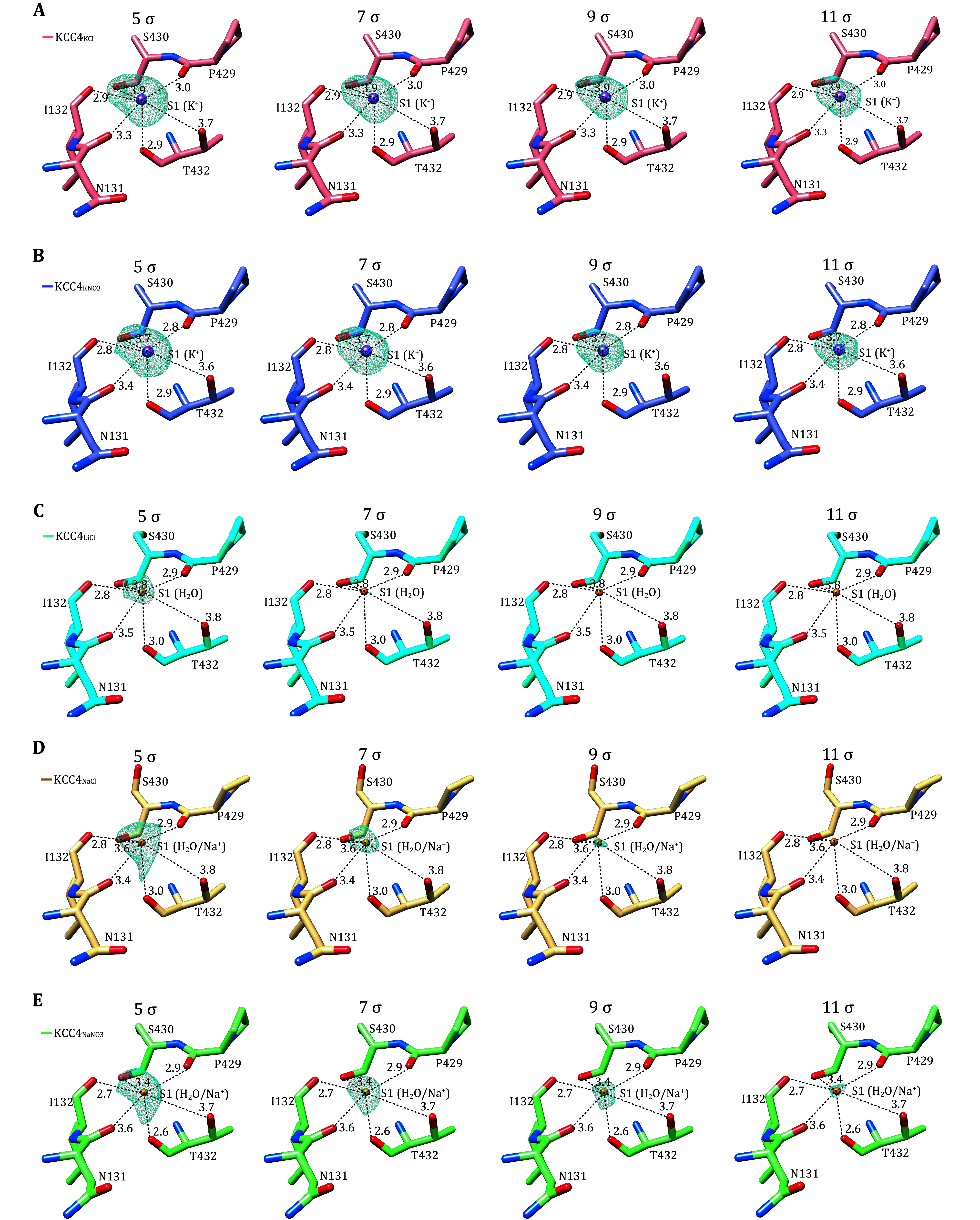
Assignment of the K^+^, H_2_O, or H_2_O/Na^+^ at S1 in KCC4_KCl_, KCC4_KNO3_, KCC4_LiCl_, KCC4_NaCl_, and KCC4_NaNO3_. **A** The K^+^ at the S1 site in the KCC4_KCl_ structure. **B** The K^+^ at the S1 site in the KCC4_KNO3_ structure. **C** The H_2_O at the S1 site in the KCC4_LiCl_ structure. **D** The H_2_O/Na^+^ at the S1 site in the KCC4_NaCl_ structure. **E** The H_2_O/Na^+^ at the S1 site in the KCC4_NaNO3_ structure. To perform the comparison, all maps are scaled and the densities are shown at the contour levels of 5 σ, 7 σ, 9 σ, and 11 σ. Dash lines show distances between K^+^, H_2_O, or H_2_O/Na^+^ and their coordination residues in Å

S1 is surrounded by five main-chain carbonyls (Asn131, Ile132, Pro429, Ser430, and Thr432) from both Gly-containing loops in TM1 and TM6 and one side-chain hydroxyl of Thr432, with coordination distances of around 2.8 to 3.9 Å (Fig. 4). This chemical environment is suitable for a cation or a water molecule, but not an anion. In the KCC4_KCl_ map, the strong density at S1 clearly indicates one K^+^ binds there (Fig. 4A). Similar strong density is also observed at S1 in the KCC4_KNO3_ map (Fig. 4B), whereas in the maps of KCC4_LiCl_, KCC4_NaCl_, and KCC4_NaNO3_, which were solved in the absence of K^+^, densities at S1 are all much weaker (Figs. 4C–4E). These data collectively demonstrate that K^+^ binds at the S1 site in KCC4_KCl_ and in KCC4_KNO3_.

Then what ions bind at S1 in KCC4_LiCl_, KCC4_NaCl_, and KCC4_NaNO3_? In KCC4_LiCl_, it is very likely that one water molecule binds at S1 in the absence of K^+^ (Creze *et al*. 2007), as we do not expect to resolve the Li^+^ ion at the current 2.49 Å resolution (Fig. 4C). Consistently, a previous functional study suggested that Li^+^ may not directly bind to the K^+^ site in KCC (Ferrell *et al*. 2000). In KCC4_NaCl_ and KCC4_NaNO3_, densities at S1 sites may arise from Na^+^ and (or) water molecules (Figs. 4D and 4E). Water and Na^+^ have similar molecular weights and are indistinguishable from density intensity at current resolutions. Here we temporarily assign H_2_O/Na^+^ (H_2_O and (or) Na^+^) at S1 sites in both KCC4_NaCl_ and KCC4_NaNO3_ (Figs. 4D and 4E).

### Coupling of K^+^ at S1 and Cl^−^ at S2

**Figure 5:**
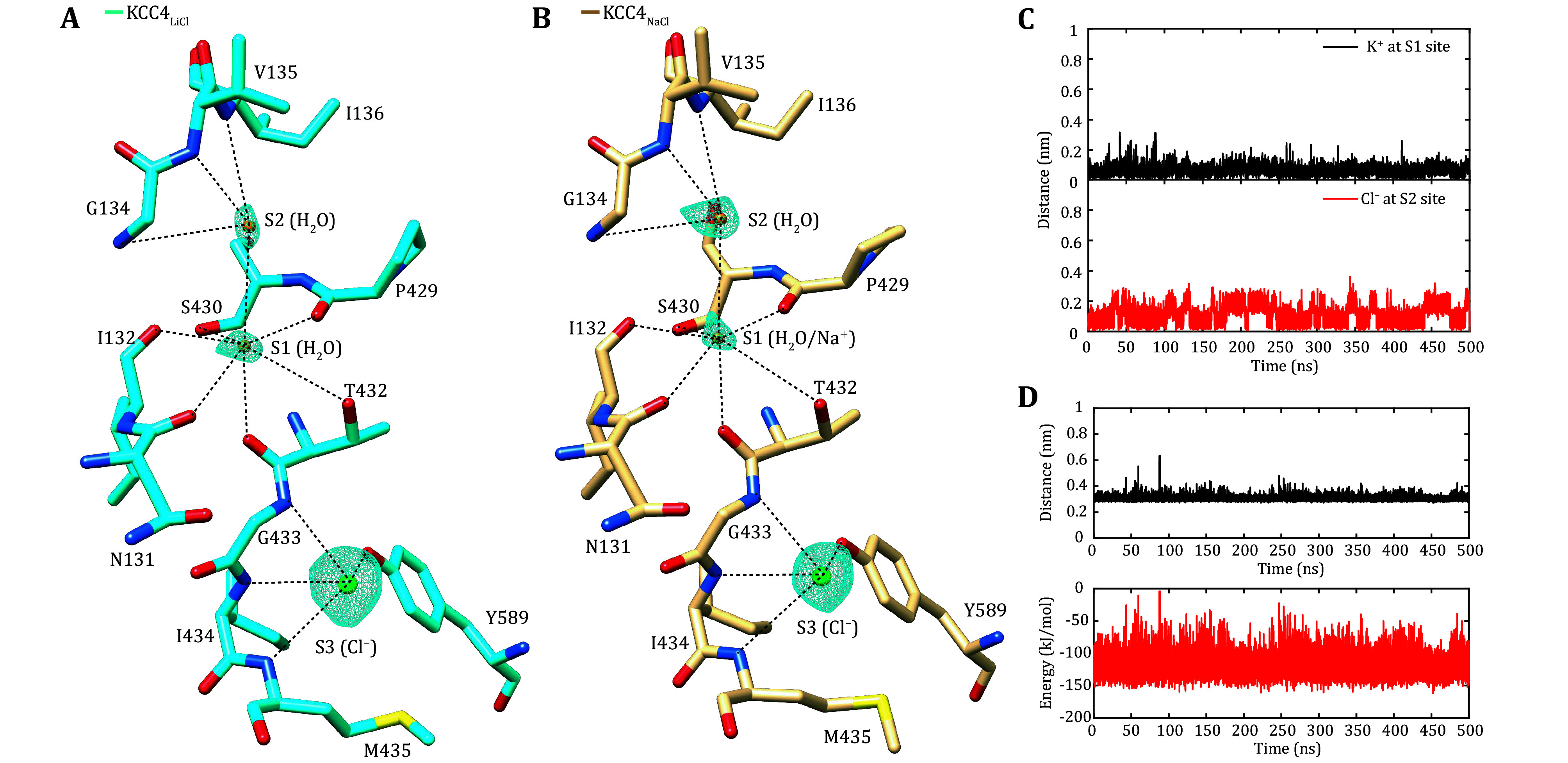
The coupling of K^+^ at S1 and Cl^−^ at S2. **A** In the KCC4_LiCl_ structure, the density of H_2_O or Cl^−^ at S1–S3 at the contour level of 5.5 σ. **B** In the KCC4_NaCl_ structure, the density of H_2_O or Cl^−^ at S1–S3 at the contour level of 8 σ. **C** Time evolution of the distances of K^+^ at S1 site (upper panel) and Cl^−^ at S2 site (lower panel) from their initial binding sites in KCC4_KCl_, respectively. **D** Time evolution of the distance (upper panel) and interaction energy (lower panel) between K^+^ at S1 site and Cl^−^ at S2 site

We further examined the ion species at S2 and S3 sites in KCC4_NaCl_ and KCC4_LiCl_. In both maps, the strong densities at S3 allow us to confidently assign Cl^−^ ions there (Figs. 5A and 5B). By contrast, in both maps of KCC4_NaCl_ and KCC4_LiCl_, densities at S2 are comparable to those at S1, but much weaker than those at S3 (Figs. 5A and 5B). Therefore, we modeled water molecules at the S2 site in both structures of KCC4_NaCl_ and KCC4_LiCl_ (Figs. 5A and 5B). Thus, in KCC4_NaCl_ and KCC4_LiCl_, in the absence of K^+^, Cl^−^ ions only bind at S3 but not at S2 although at a high concentration.

Now we can summarize ions bound at S1, S2, and S3 sites in the five KCC4 structures, namely KCC4_KCl_, KCC4_KNO3_, KCC4_NaNO3_, KCC4_LiCl_, and KCC4_NaCl_ (Table 1). The binding of K^+^ at S1 is independent of Cl^−^ binding at S2 and (or) at S3, as revealed by the structure of KCC4_KNO3_ (Figs. 2B, 3B, and 4B). Similarly, the binding of Cl^−^ at S3 is also independent of K^+^ at S1, as shown in the structures of KCC4_LiCl_ and KCC4_NaCl_ (Figs. 5A and 5B). By contrast, the binding of Cl^−^ at S2 depends on the K^+^ at S1. In KCC4_LiCl_ and KCC4_NaCl_, Cl^−^ can not bind at S2 in the absence of K^+^ at S1 (Figs. 5A and 5B). Therefore, Cl^−^ at S2 is tightly coupled to K^+^ at S1. Such a coupling of Cl^−^ and K^+^ is largely attributed to their direct interaction (Fig. 2A). More importantly, the coupling of Cl^−^ at S2 to K^+^ at S1 strongly supports that the Cl^−^ at S2 instead of S3 is the cotransported Cl^−^ in KCCs because dissociation of K^+^ at S1 will inevitably result in the concurrent release of Cl^−^ from S2, as shown in the structures of KCC4_LiCl_ and KCC4_NaCl_ (Figs. 5A and 5B).

**Table 1:** Ions bound at S1–S3 sites in six KCC4 structures

Structures	KCC4_KCl_	KCC4_KNO3_	KCC4_NaNO3_	KCC4_LiCl_	KCC4_NaCl_	KCC4_10RbCl_
S1	K^+^	K^+^	H_2_O/Na^+^	H_2_O	H_2_O/Na^+^	Rb^+^
S2	Cl^−^	H_2_O	H_2_O	H_2_O	H_2_O	Cl^−^
S3	Cl^−^	H_2_O	H_2_O	Cl^−^	Cl^−^	Cl^−^

To further validate the coupling of K^+^ at S1 and Cl^−^ at S2, we then performed molecular dynamics (MD) simulation of KCC4_KCl_ under 150 mmol/L KCl system in the lipid bilayer using the Orientations of Proteins in Membranes (OPM) method (Lomize *et al*. 2006) and CHARMM-GUI software packages (Jo *et al*. 2008). In an equilibrium state without an electrochemical gradient, the K^+^ and Cl^−^ remained stably bound at the S1 and S2 in KCC4_KCl_ throughout 500-ns simulation, with distances to their initial positions below 2 Å, respectively (Fig. 5C). Besides, the two ions form strong interactions, with their coordination distance less than 3.5 Å and interaction energy stronger than –100 kJ/mol (Fig. 5D). These results support the coupled binding of K^+^ at S1 and Cl^−^ at S2.

### The structural basis for K^+^ selectivity

**Figure 6:**
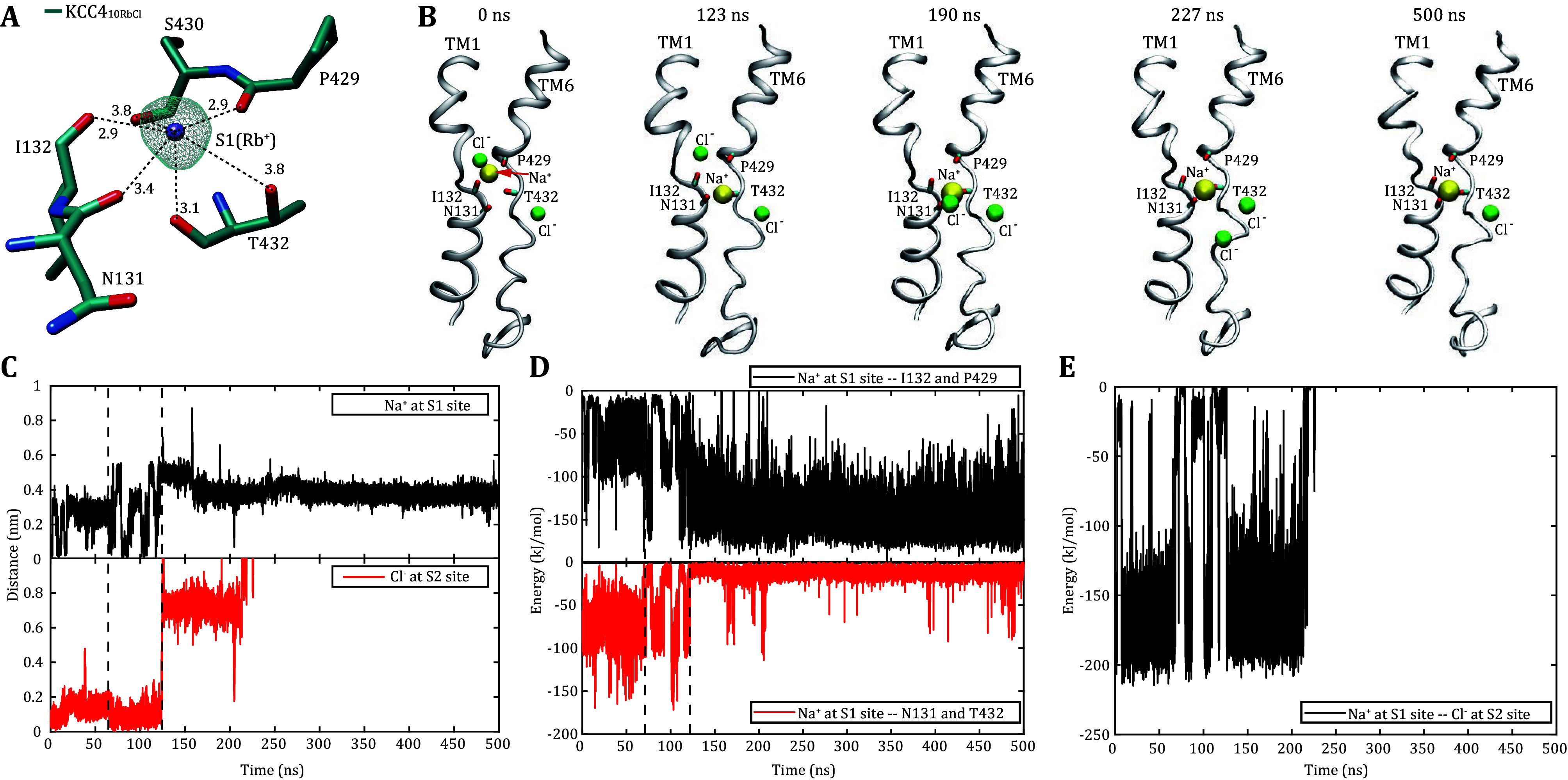
Selectivity of K^+^ over Na^+^ at S1 site. **A** In KCC4_10RbCl_ structure, the density of Rb^+^ at S1 at the contour level of 10 σ. **B** Representative states of ion binding sites in KCC4 during the MD simulation in 150 mM NaCl system, with Na^+^, Cl^−^ and Cl^−^ originally bound at S1–S3, respectively. Five snapshots were captured at the time course of 0 ns, 123 ns, 190 ns, 227 ns, and 500 ns. **C** Time evolution of the distances of Na^+^ at S1 site (upper panel) and Cl^−^ at S2 site (lower panel) from their original binding sites, respectively. **D** Time evolution of interaction energy between Na^+^ at S1 and carbonyl groups of I132 and P429 (upper panel), and between Na^+^ at S1 and two carbonyl groups of N131 and T432 (lower panel). **E** Time evolution of interaction energy between Na^+^ at the S1 site and Cl^−^ at the S2 site

To reveal the structural basis of K^+^ selectivity, we also determined the KCC4_10RbCl_ structure in the presence of 140 mmol/L NaCl and 10 mmol/L RbCl. Rb^+^ has a similar radius as K^+^ and is often used as a substitute for K^+^ in functional assays and structural determination of potassium channels and transporters (Miyamoto *et al*. 1978; Morais-Cabral *et al*. 2001). In the KCC4_10RbCl_ map, strong density at S1 indicates that Rb^+^ can effectively bind there even in the low concentration (Fig. 6A), whereas neither water nor Na^+^ can compete with Rb^+^ at this site. Thus, the S1 site exhibits remarkable selectivity of Rb^+^ (K^+^) over water and Na^+^.

The configuration of the S1 site and the coordination distances of K^+^ at S1 provide clues for the K^+^ selectivity of KCC4. In the KCC4_KCl_ structure, the K^+^ at S1 is mainly coordinated by four main-chain carbonyls of Asn131, Ile132, Pro429, and Thr432, arranged in a tetrahedron configuration. The average coordination distance of these four main interactions is 2.99 ± 0.20 Å (Fig. 4A), similar to the coordination distances of dehydrated K^+^ ions bound in the selectivity filter of the potassium channel such as KcsA and MthK (Morais-Cabral *et al*. 2001; Ye *et al*. 2010). In KCC4_LiCl_, KCC4_NaCl_, and KCC4_NaNO3,_ the average coordination distance of the four main-chain carbonyls to the H_2_O or H_2_O/Na^+^ at S1 is 3.08 ± 0.29 Å, 3.04 ± 0.25 Å, and 2.94 ± 0.43 Å, respectively (Figs. 4C–4E). Thus, when a Na^+^ or a water molecule binds at S1, it tends to move away from the center of the tetrahedron S1 site to adjust the coordination distances due to its smaller dehydration radius, resulting in a larger average or derivation of the coordination distance.

However, due to the resolution limit, the measurements of coordination distances of ions at the S1 site may not be very accurate. To verify the hypothesis of ion selectivity in KCCs, we performed MD simulations by placing Na^+^ and Cl^−^ at S1 and S2 sites, respectively. As the simulation proceeds, the Na^+^ first moves up to the carbonyls of Ile132 and Pro429 then drifts to a lower position closer to the carbonyls of Asn131 and Thr432, with distance to its initial position up to 4 Å (Figs. 6B and 6C). The calculated binding energies of Na^+^ to the carbonyls of Ile132 and Pro429 as well as to the carbonyls of Asn131 and Thr432 show that the Na^+^ mainly interacts with the carbonyls of Asn131 and Thr432 rather than Ile132 and Pro429 carbonyls after *t* = 124 ns (Fig. 6D). Thus, this simulation supports that Na^+^ at S1 tends to move away from the center because of its smaller dehydration radius and coordination distances.

The drift of Na^+^ to Asn132 and Thr432 carbonyls (after *t* = 124 ns) induces the loss of the interaction between Na^+^ and Cl^−^ (Fig. 6E) and the dissociation of Cl^−^ from the S2 site (Figs. 6B and 6C). Although the Cl^−^ at S2 is also coordinated by three main chain amide groups of Gly134, Val135, and Ile136 (Fig. 2A), the cation at S1 seems to play a dominant role in the binding stability of Cl^−^. K^+^ with a suitable radius not only binds stably at S1 but also provides strong coordination for the Cl^−^ at S2, whereas Na^+^ tends to move away from the Cl^−^ at S2 and consequently disrupts their coordination interaction, resulting in the dissociation of Cl^−^ from S2 site (Figs. 6B–6E). This simulation reveals the structural basis of the ion selectivity and supports the coupled release of K^+^ from S1 and Cl^−^ from S2.

### The possibility of Na^+^ binding at S1

As demonstrated by previous studies, KCCs neither transport Na^+^ nor are inhibited by Na^+^ (Delpire and Lauf 1991; Kakazu *et al*. 2000; Williams and Payne 2004). Under physiological conditions with a high concentration of K^+^ and a low concentration of Na^+^ in the cytosol, Na^+^ should not bind at S1 otherwise it would inhibit the K-Cl cotransport activity of KCCs. However, we are not sure whether Na^+^ is able to bind at S1 in the absence of K^+^ in our structures of KCC4_NaCl_ and KCC4_NaNO3_. The MD simulation of KCC4 with Na^+^ at S1 suggests that the smaller Na^+^ binds at the tetrahedron S1 site with limited stability and moves closer to Asn131 and Thr432 (Figs. 6B–6D). This Na^+^ can not compete with K^+^ (or Rb^+^) at S1, even at a low concentration of K^+^ (or Rb^+^), as demonstrated by the structure of KCC4_10RbCl_ (Fig. 6A). Taken together, we propose that the S1 site displays a much higher affinity of K^+^ over Na^+^, which can explain no detectable inhibition effect of Na^+^ on KCCs in previous transport assays (Delpire and Lauf 1991; Kakazu *et al*. 2000; Williams and Payne 2004).

### Comparison with K^+^ selectivity of potassium channels

In classical K^+^-selective channels such as KcsA and MthK, the K^+^ selectivity is determined by the elegant K^+^ selectivity filter, which contains four K^+^ binding sites (Doyle *et al*. 1998; Ye *et* *al*. 2010). The 2.7–3.0 Å coordination distances at each site perfectly match the radius of dehydrated K^+^, but are larger than that of Na^+^ (supplementary Fig. S8). Potassium channels usually have two sites with high affinity to K^+^, which ensures that K^+^ at low concentration can compete with Na^+^ (Sauer *et al*. 2013; Ye *et al*. 2010). The S1 site in KCC4 may share similar features with the K^+^ sites in the selectivity filter in potassium channels although their chemical environments and coordination numbers are not identical. In KCC4, the S1 site is designed for K^+^ that has a matched dehydration radius and therefore displays a higher affinity to K^+^ than Na^+^. To achieve a high conductance, potassium channels need to maintain four K^+^ sites at the selectivity filter (Kopfer *et al*. 2014), while in KCCs one K^+^ site seems sufficient for the selective transport of K^+^.

### The K-Cl cotransport mechanism

KCCs cotransport K^+^ and Cl^−^ at a 1:1 ratio (Jennings and Adame 2001). The observation of two Cl^−^ sites along with one K^+^ in KCC1 (Liu *et al*. 2019) and KCC4 (Xie *et al*. 2020) is very puzzling in light of the strict electroneutrality of K-Cl cotransport. In this report, using cryo-EM titration and computational study, we have revealed the coupled binding and release of K^+^ at S1 and Cl^−^ at S2, suggesting that the Cl^−^ at S2 but not at S3 is the cotransported one. As both Cl^−^ sites S2 and S3 are conserved among CCC family (Chew *et al*. 2019; Fan *et al*. 2023; Liu *et al*. 2019; Nan *et al*. 2022; Xie *et al*. 2020) and NKCCs cotransport both Cl^−^ ions with one Na^+^ and one K^+^ in one cycle, it is interesting whether the Cl^−^ at S3 in KCCs is also released at any point of the transport cycle. Could it be that this Cl^−^ is actually released in outside-facing conformation, but its re-binding is necessary for KCCs to return to inward-facing conformation to complete a transport cycle? In this case, the function of KCCs would be equivalent to K-Cl cotransport plus Cl^−^/Cl^−^ exchange. This transport modality has not been described in the literature but would certainly satisfy the electroneutrality requirement. Alternatively, is it possible that KCCs cotransport two K^+^ and two Cl^−^ in one cycle, with one K^+^ replacing the Na^+^ in NKCCs? In fact, no K^+^ density was observed in the site in KCC1 corresponding to the Na^+^ site in NKCC1, which largely rules out this possibility (Liu *et al*. 2019).

The relative positions of one K^+^ and two Cl^−^ sites in the substrate-binding sites of KCCs are also interesting in light of previous data showing ordered binding for influx with Cl^−^ first, followed by K^+^ (Fig. 1C) (Delpire and Lauf 1991). Because S3 is relatively deep within the permeation pathway compared to S1 and S2, it is possible that the binding of Cl^−^ at S3 from the outside must precede the binding of K^+^ and Cl^−^ at S1 and S2, respectively. In contrast, as the internal cavity is larger, the movement of K^+^ towards S1 and Cl^−^ towards S2 or S3 might not be prevented by the interaction of the counter-ion at its binding site, thereby allowing random binding (Delpire and Lauf 1991). Note that for this model to work, the interaction of Cl^−^ at S3 needs to satisfy rapid equilibrium kinetics, *i*.*e*. binding and release of Cl^−^ at S3 being fast reactions compared to conformational changes of the transporter. Altogether, our cryo-EM data bring clarity as to the specificity and selectivity of the ion-binding sites but they also challenge our understanding of stoichiometry and ion binding modalities for K-Cl cotransport.

## MATERIALS AND METHODS

### Protein expression and purification

Human KCC4a (Uniprot code: Q9H2X9-1) were cloned, expressed, and purified following similar procedures in our previous study (Xie *et al*. 2020). Briefly, human KCC4a was heterologously expressed in HEK293F cells (Life Technologies) using the BacMam system (Thermo Fisher Scientific). The baculovirus generated in Sf9 cells (Life Technologies) following the standard protocol was used to infect HEK293F cells at a ratio of 1∶20 (virus: HEK293F, *v*∶*v*), supplemented with 10 mmol/L sodium butyrate to boost protein expression. Cells were cultured in suspension at 37°C for 48 h and then harvested by centrifugation at 3000*g*.

To prepare KCC4 samples in different ion conditions, we used different buffer solutions in protein purification. For the 150 mmol/L KCl condition, we followed the exact same procedure as before. For 140 mmol/L NaCl + 10 mmol/L RbCl condition, we replaced 150 mmol/L KCl with 140 mmol/L NaCl + 10 mmol/L RbCl throughout the purification. For 150 mmol/L LiCl or 150 mmol/L NaCl condition, LiCl or NaCl was the only salt in the buffer. For the 150 mmol/L KNO_3_ or 150 mmol/L NaNO_3_ system, KNO_3_ or NaNO_3_ was the only salt in the buffer. In each condition, the cell pellet was re-suspended in Buffer A (30 mmol/L Tris-HCl pH 8.0 for LiCl, NaCl, KCl, and NaCl + RbCl conditions, or 30 mmol/L Tris-HNO_3_ pH 8.0 for KNO_3_, NaNO_3_ conditions, and 150 mmol/L corresponding salt) supplemented with a protease inhibitor cocktail (2 μg/mL pepstatin, 2 μg/mL leupeptin and 2 μg/mL aprotinin, and 1 mmol/L PMSF) and 2 μg/mL DNase I, and homogenized by sonication on ice. KCC4 was extracted with 1% (*w:v*) lauryl maltose neopentyl glycol (LMNG, Anatrace) supplemented with 0.2% (*w:v*) cholesteryl hemisuccinate (CHS, Sigma Aldrich) by gentle agitation for 2 h at 4°C. After extraction, the supernatant was collected after a 60-min centrifugation at 48,000*g* and incubated with Anti-DYKDDDDK G1 Affinity Resin (GenScript) with gentle agitation. After 2 h, the resin was collected on a disposable gravity column (Bio-Rad) and washed in Buffer B (Buffer A + 0.005% LMNG + 0.02% CHS) for ten column volumes. The detergent was then changed to 0.02% glycol-diosgenin (GDN) and the protein sample was eluted by 0.2 mg/mL FLAG peptide in Buffer C (Buffer A + 0.02% GDN). The protein sample was further purified by size exclusion chromatography on a Superose 6 10/300 GL column (GE Healthcare) pre-equilibrated with Buffer C. The protein peak fractions were collected and concentrated to ~30–40 mg/mL for cryo-EM analysis.

### EM data acquisition

The cryo-EM grids were prepared by applying 3 μL KCC4 protein to a glow-discharged Quantifoil R1.2/1.3 200 mesh copper/gold holey carbon grid (Quantifoil, Micro Tools GmbH, Germany) and blotting for 3.0 s under 100% humidity at 4°C before being plunged into liquid ethane using a Mark IV Vitrobot (Thermo Fisher Scientific). Micrographs were acquired on a Titan Krios microscope (Thermo Fisher Scientific) operated at 300 kV with a K3 Summit direct electron detector and a bioquantum energy filter at a slit width of 20 eV (Gatan). EPU software was used for automated data collection following the standard procedure. The data was collected in super-resolution mode with a pixel size of 0.4255 Å. The defocus range was set from −1.1 to −1.5 μm. Each micrograph was dose-fractionated to 40 frames under a dose rate of 15.658 e^−^/(pixel·s), with a total exposure time of 2.5 s, resulting in a total dose of about 54 e^−^/Å^2^.

### Image processing

The motion correction was performed with the MotionCor2 program (Zheng *et al*. 2017) or RELION (Scheres 2012) and the CTF parameters of the micrographs were estimated with the GCTF program (Zhang 2016). All other steps of image processing were performed in RELION.

Laplacian-of-Gaussian-based auto-picking of RELION was used for particle picking (Scheres 2012). A total of 2,113,110, 2,833,122, 2,591,719, 3,035,174, 16,477,001, and 2,635,282 particles were picked from 3340 images of KCC4_KCl_, 3083 images of KCC4_LiCl_, 3990 images of KCC4_NaCl_, 4528 images of KCC4_10RbCl_, 2783 images of KCC4_KNO3_, and 3823 images of KCC4_NaNO3_, respectively. After particle extraction, with or without 2D classification, two or three rounds of 3D classification were performed using the previously reported KCC4 structure (KCC4_7D99_) as a reference. *C2* symmetry was applied for all datasets during the 3D classification and refinement. After polishing with RELION, the resulting 3D reconstruction of KCC4 for different datasets yielded an EM map with a resolution of 2.49 Å for KCC4_KCl_, 2.49 Å for KCC4_LiCl_, 2.58 Å for KCC4_NaCl_, 2.45 Å for KCC4_10RbCl_, 2.38 Å for KCC4_KNO3_, and 2.40 Å for KCC4_NaNO3_. The resolution was estimated by applying a soft mask around the protein density and the gold-standard Fourier shell correlation (FSC) = 0.143 criterion. Local resolution maps were calculated with RELION (Scheres 2012).

### Cryo-EM map normalization

For the comparison of different cryo-EM maps, the other five maps were normalized to the KCC4_KNO3_ map in Chimera (Pettersen *et al*. 2004). Briefly, the other five maps were first resampled on the KCC4_KNO3_ map and then scaled based on their scaling factors. The calculation formula is that the scaling factor equals the root mean square (RMS) of KCC4_KNO3_ divided by that of KCC4_X_, where subscript X represents LiCl, NaCl, KCl, 10RbCl, and NaNO_3_. The RMSD values of KCC4_KNO3_, KCC4_LiCl_, KCC4_NaCl_, KCC4_KCl_, KCC4_10RbCl_, and KCC4_NaNO3_ are 0.0059978, 0.0025933, 0.0047341, 0.0049554, 0.0040755, and 0.0047008, respectively. Consequently, the scaling factors of KCC4_LiCl_, KCC4_NaCl_, KCC4_KCl_, KCC4_10RbCl_, and KCC4_NaNO3_ to KCC4_KNO3_ are 2.3128, 1.2669, 1.2104, 1.4717, and 1.2759, respectively.

### Model building, refinement, and validation

Atomic model building based on the 2.9 Å resolution structure of KCC4 (PDB: 7D99) was performed in Coot (Emsley *et al*. 2010; Xie *et al*. 2020). Models were refined against summed maps using Phenix.real_space_refine (Adams *et al*. 2010), with secondary structure restraints and non-crystallography symmetry applied. The statistics for the models’ geometries were generated by MolProbity (Chen *et al*. 2010). All Figures were prepared in PyMol (Schrodinger 2015) or Chimera (Pettersen *et al*. 2004).

### MD simulation

The KCC4 dimer was embedded into the lipid bilayer composed of 320 POPC molecules by using the Orientations of Proteins in Membranes (OPM) method (Lomize *et al*. 2006) and CHARMM-GUI software packages (Jo *et al*. 2008). The titratable residues were set to their dominant protonation state at pH 7.0. The system was then solvated by water. The box size was 12 nm × 12 nm × 16.5 nm, which contains ~230,000 atoms. The parameters for protein, lipid, and ions were taken from CHARMM36 force field (Huang and MacKerell 2013). The TIP3P model was chosen for water molecules (Jorgensen *et al*. 1983). ~150 positive ions and ~150 negative ions were added to neutralize the system and bring its total ionic strength to 150 mmol/L. We first considered the system wherein three sites (*i*.*e*., S1, S2, and S3) were initially occupied by K^+^, Cl^−^, and Cl^−^ in 150 mmol/L KCl solution which was denoted as sys_KCl. To study the ion selectivity at S1, we constructed another system sys_NaCl, wherein the S1 site was initially occupied by Na^+^ and S2/S3 sites were initially occupied by Cl^−^ in 150 mmol/L NaCl solution.

Each constructed system was first minimized using the steepest descent for 5000 steps, then 250 ps of NVT and 12 ns of NPT equilibration. During the NPT equilibration positional restraints on the heavy atoms of lipids, protein side chains, and protein backbone atoms were gradually decreased. For both sys_KCl and sys_NaCl, six independent 500-ns simulations were performed. A typical 1.2-nm cutoff distance was used to calculate the short-range electrostatic interaction as well as the van der Waals interaction. The particle mesh Ewald (PME) method was employed to compute the long-range electrostatic interaction. The periodic boundary conditions were applied in all directions. The system temperature and pressure were controlled using the velocity-rescaled Nose-Hoover thermostat and the semi-isotropic Parrinello-Rahman barostat respectively. The LINCS algorithm was adopted to constrain the bond vibrations involving hydrogen atoms, allowing a time step of 2 fs. All MD simulations were carried out using the GROMACS 5.1.2 package (Hess *et al*. 2008) and snapshots were rendered by the visual molecular dynamics (VMD) program (Humphrey *et al*. 1996). To quantify the distance of ions from their corresponding binding site, a set of residue atoms were selected whose center of mass represents the position of binding site: backbone carbonyl oxygen atoms of Asn131, Ile132, Pro429, and Thr432 (S1 site); backbone amide nitrogen atoms of Val135 and Ile136 (S2 site).

## Data Availability

Cryo-EM density maps have been deposited in the Electron Microscopy Data Bank (EMDB) under accession numbers 33090 for KCC4_KCl_, 33092 for KCC4_LiCl_, 33093 for KCC4_NaCl_, 33095 for KCC4_10RbCl_, 33091 for KCC4_KNO3_, 33094 for KCC4_NaNO3_. Structure coordinates have been deposited in the protein data bank (PDB) under accession numbers 7XAG for KCC4_KCl_, 7XAI for KCC4_LiCl_, 7XAJ for KCC4_NaCl_, 7XAL for KCC4_10RbCl_, 7XAH for KCC4_KNO3_, 7XAK for KCC4_NaNO3_.
